# NH_2_OH Disproportionation Mediated by Anaerobic Ammonium-oxidizing (Anammox) Bacteria

**DOI:** 10.1264/jsme2.ME21092

**Published:** 2022-04-12

**Authors:** Mamoru Oshiki, Lin Gao, Lei Zhang, Satoshi Okabe

**Affiliations:** 1 Division of Environmental Engineering, Faculty of Engineering, Hokkaido University, Sapporo, Japan

**Keywords:** anammox bacteria, hydroxylamine (NH_2_OH), NH_2_OH disproportionation, ^15^N-tracing technique, up-flow column reactor

## Abstract

Anammox bacteria produce N_2_ gas by oxidizing NH_4_^+^ with NO_2_^–^, and hydroxylamine (NH_2_OH) is a potential intermediate of the anammox process. N_2_ gas production occurs when anammox bacteria are incubated with NH_2_OH only, indicating their capacity for NH_2_OH disproportionation with NH_2_OH serving as both the electron donor and acceptor. Limited information is currently available on NH_2_OH disproportionation by anammox bacteria; therefore, the stoichiometry of anammox bacterial NH_2_OH disproportionation was examined in the present study using ^15^N-tracing techniques. The anammox bacteria, *Brocadia sinica*, *Jettenia caeni*, and *Scalindua* sp. were incubated with the addition of ^15^NH_2_OH, and the production of ^15^N-labeled nitrogenous compounds was assessed. The anammox bacteria tested performed NH_2_OH disproportionation and produced ^15-15^N_2_ gas and NH_4_^+^ as reaction products. The addition of acetylene, an inhibitor of the anammox process, reduced the activity of NH_2_OH disproportionation, but not completely. The growth of *B. sinica* by NH_2_OH disproportionation (–240.3‍ ‍kJ mol NH_2_OH^–1^ under standard conditions) was also tested in 3 up-flow column anammox reactors fed with 1) 0.7‍ ‍mM NH_2_OH only, 2) 0.7‍ ‍mM NH_2_OH and 0.5‍ ‍mM NH_4_^+^, and 3) 0.7‍ ‍mM NH_2_OH and 0.5‍ ‍mM NO_2_^–^. NH_2_OH consumption activities were markedly reduced after 7‍ ‍d of operation, indicating that *B. sinica* was unable to maintain its activity or biomass by NH_2_OH disproportionation.

Anaerobic ammonium-oxidizing (anammox) bacteria were initially discovered in a denitrifying bioreactor in 1995 (Mulder *et al.*, 1995), and are now recognized as the main players in the global nitrogen cycle (Kuypers *et al.*, 2003; 2005; Amano *et al.*, 2007; Yoshinaga *et al.*, 2011). In the anammox process, NH_4_^+^ is oxidized to N_2_ gas using NO_2_^–^ as an electron acceptor, and the stoichiometry of the anammox process has been described as follows (Lotti *et al.*, 2014):

1 NH_4_^+^+1.146 NO_2_^–^+0.071 HCO_3_^–^+0.057 H^+^

→ 0.986 N_2_+0.161 NO_3_^–^+0.071 CH_1.74_O_0.31_N_0.20_+2.002 H_2_O eq. 1

As an intermediate, hydrazine (N_2_H_4_) is synthesized from NH_4_^+^ and NO or NH_2_OH by hydrazine synthase, and its biosynthesis appears to be unique to metabolism by anammox bacteria (Strous *et al.*, 2006; Kartal *et al.*, 2011; Oshiki *et al.*, 2016a). Anammox bacteria are monophyletically affiliated into the bacterial order *Brocadiales* in the phylum *Planctomycetota* (Strous *et al.*, 1999), and five candidate genera have been proposed: *Candidatus Brocadia*, *Kuenenia*, *Jettenia*, *Anammoxoglobus*, and *Scalindua* (Egli *et al.*, 2003; Kuypers *et al.*, 2003; Quan *et al.*, 2008). These anammox bacteria have been detected in various natural and man-made ecosystems and are significantly involved in nitrogen loss (Oshiki *et al.*, 2016b).

Hydroxylamine (NH_2_OH), a well-known intermediate of the aerobic NH_3_ oxidation reaction (Madigan *et al.*, 2019), is also a potential intermediate of the anammox process (van de Graaf *et al.*, 1997). The anammox bacterium, *Brocadia sinica* reduced NO_2_^–^ to NH_2_OH (Oshiki *et al.*, 2016a) potentially by using reductive hydroxylamine dehydrogenase (rHao) (Ferousi *et al.*, 2021), and synthesized N_2_H_4_ from the NH_2_OH and NH_4_^+^ using hydrazine synthase (Oshiki *et al.*, 2016a). *Kuenenia stuttgartiensis* reduced NO_2_^–^ to NO (Kartal *et al.*, 2011), and NO was further reduced to NH_2_OH by hydrazine synthase to synthesize N_2_H_4_ (Dietl *et al.*, 2015). Apart from the anammox process, the nitrogen transformation reaction, NH_2_OH disproportionation, has been reported in anammox bacteria (van der Star *et al.*, 2008). A disproportionation reaction is a reaction in which a chemical compound serves as both an electron donor and accepter, and the disproportionation reaction of inorganic sulfur (Finster, 2008) is an example of a microbial disproportionation reaction. In NH_2_OH disproportionation, NH_2_OH is converted to N_2_ gas and NH_4_^+^ using the following stoichiometry (Pacheco *et al.*, 2011):

3 NH_2_OH+H^+^ → NH_4_^+^+N_2_+3 H_2_O eq. 2

NH_2_OH disproportionation involves the following 2 reactions, N_2_H_4_ production and consumption (Soler-Jofra *et al.*, 2020):

NH_4_^+^+NH_2_OH → N_2_H_4_+H_2_O+H^+^ eq. 3

2 NH_2_OH+N_2_H_4_+2 H^+^ → 2 NH_4_^+^+N_2_+2 H_2_O eq. 4

Although NH_2_OH disproportionation by *K. stuttgartiensis* (van der Star *et al.*, 2008; Soler-Jofra *et al.*, 2020) and *B. sinica* (Oshiki *et al.*, 2016a) has been described, further studies are required to obtain a more detailed understanding of anammox bacterial NH_2_OH disproportionation for the following reasons. Although the kinetics of NH_2_OH disproportionation have been investigated using *K. stuttgartiensis* (van der Star *et al.*, 2008; Soler-Jofra *et al.*, 2020), the amounts of N_2_ gas produced in NH_2_OH disproportionation (see eq. 2) were not measured in previous studies and the stoichiometry of NH_2_OH disproportionation was not established. Furthermore, we previously examined NH_2_OH disproportionation by *B. sinica*; however, we only reported the occurrence of NH_2_OH disproportionation (Oshiki *et al.*, 2016a) and did not investigate the effects of NH_2_OH concentrations on NH_2_OH disproportionation by repeating batch incubations. In addition, although eq. 2 yields –‍240.3‍ ‍kJ mol NH_2_OH^–1^ of free energy under standard conditions (Soler-Jofra *et al.*, 2020), the growth of anammox bacteria with NH_2_OH disproportionation has not yet been examined.

Therefore, the present study investigated anammox bacterial NH_2_OH disproportionation. The phylogenetically different anammox bacteria, *B. sinica*, *Jettenia caeni*, and *Scalindua* sp. were incubated with ^15^N-labeled NH_2_OH, and the stoichiometry of NH_2_OH disproportionation was carefully assessed based on measurements of ^15-15^N_2_ and NH_4_^+^ concentrations. NH_2_OH disproportionation was also analyzed under acetylene inhibition conditions. Acetylene is a strong inhibitor of aerobic NH_3_ oxidation, N_2_O reduction to N_2_ (*i.e.*, denitrification), and the anammox process (Jensen *et al.*, 2007); however, its effects on NH_2_OH disproportionation currently remain unknown. *B. sinica* was cultured in up-flow column reactors with the addition of NH_2_OH to establish whether it grows on NH_2_OH, and the activity of NH_2_OH consumption and the abundance of the anammox bacterial 16S rRNA gene were evaluated.

## Materials and Methods

### Anammox bacterial cultures

Planktonic cells of *B. sinica*, *J. caeni*, and *Scalindua* sp. were cultivated in membrane bioreactors (MBRs) equipped with a hollow fiber membrane module (pore size of 0.1‍ ‍μm, polyethylene) as previously described (Oshiki *et al.*, 2013; Zhang and Okabe, 2020). Culture media fed into MBRs contained KH_2_PO_4_ (24.4‍ ‍mg L^–1^), MgSO_4_·7H_2_O (60‍ ‍mg L^–1^), CaCl_2_ (51‍ ‍mg L^–1^), yeast extract (Becton, Dickinson and Company) (1.0‍ ‍mg L^–1^), and 0.5‍ ‍mL of trace element solutions I and II (van de Graaf *et al.*, 1996). The artificial sea salt SEALIFE (Marine Tech) (Kindaichi *et al.*, 2011) was supplemented into media for “*Ca.* Scalindua sp.” at a final concentration of 28‍ ‍g L^–1^. Equimolar amounts of NH_4_(SO_4_)_2_ and NaNO_2_ were supplemented into media at 10‍ ‍mM for *B. sinica* and *Scalindua* sp. and 5‍ ‍mM for *J. caeni*, and the nitrogen loading rates of the MBRs for *B. sinica*, *J. caeni*, and *Scalindua* sp. were set at 0.55, 0.18, and 0.45‍ ‍kg N m^–3^ d^–1^, respectively. MBRs were operated at 37°C for *B. sinica* and at 25°C for *J. caeni* and *Scalindua* sp. pH was not controlled in MBRs, but was in the range of pH 7.6–8.0. Anammox bacterial cells accounted for more than 90% of the total biomass in MBRs as measured by a fluorescence *in-situ* hybridization (FISH) ana­lysis using the oligonucleotide probes AMX820 (Schmid *et al.*, 2001) and EUBmix composed of equimolar EUB338, EUB338II, and EUB338III (Daims *et al.*, 1999). Anammox bacterial species were routinely checked based on the partial anammox bacterial 16S rRNA gene sequence using Sanger sequencing (Oshiki *et al.*, 2011).

### Batch incubations of anammox bacteria

Standard anaerobic techniques were employed in an anaerobic chamber (Coy Laboratories Products) in which the concentration of oxygen was maintained at <1 ppm. Culture media and stock solutions were prepared by purging N_2_ gas for >30‍ ‍min, and then repeatedly vacuuming and purging He gas. The ^15^N enrichment of ^15^NH_2_OH·HCl (Cambridge Isotope Laboratories) was >98%.

Anammox bacterial cells collected from MBRs were centrifuged at 13,420×*g* at 20°C for 10‍ ‍min, washed, and then resuspended in the above culture media without NH_4_^+^ and NO_2_^–^ at concentrations of 0.5‍ ‍mg protein mL^–1^. Twenty-five milliliters of the cell suspension was dispensed into 70-mL serum glass vials (Nichiden-Rika glass), and the headspace was replaced with He gas (>99.99995%) after sealing with butyl rubber stoppers and aluminium caps. Vials were incubated after the addition of ^15^NH_2_OH (final concentration of 1.0 to 10‍ ‍mM) and acetylene (30‍ ‍μM) (Jensen *et al.*, 2007) in the dark at 37°C for *B. sinica* and at 25°C for *J. caeni* and *Scalindua* sp.. Liquid samples were collected using a 1-mL plastic disposable syringe, immediately filtered using a 0.2-μm cellulose acetate filter, and subjected to measurements of NH_4_^+^, NO_2_^–^, NO_3_^–^, and NH_2_OH concentrations. Gas samples were collected using a gas-tight glass syringe and immediately injected into a gas chromatograph to assess ^14-15^N_2_ and ^15-15^N_2_ concentrations.

### Up-flow column reactors fed with NH_2_OH

Three 255-mL up-flow column reactors were operated at 37°C in the dark with the continuous feeding of the above culture media containing 1) 0.7‍ ‍mM ^14^NH_2_OH, 2) 0.7‍ ‍mM ^14^NH_2_OH and 0.5‍ ‍mM ^14^NH_4_^+^, or 3) 0.7‍ ‍mM ^14^NH_2_OH and 0.5‍ ‍mM ^14^NO_2_^–^. *B. sinica* cells immobilized on polyvinyl alcohol (PVA)-sodium alginate (SA) (6 and 2% [w/v], respectively) beads were inoculated into the column reactors at a packing ratio of 50% (v/v). The gel immobilization of *B. sinica* cells in PVA-SA gel beads was performed as previously described (Ali *et al.*, 2015). Briefly, the planktonic cells of *B. sinica* collected from the above MBR were resuspended in culture media, and mixed with an equal volume of PVA-alginate (FUJIFILM Wako) solution (12 and 4% [w/v], respectively). The gel solution was dropped using a disposable 50-mL plastic syringe (Terumo) into a 4% (w/v) CaCl_2_ solution to form gel beads (diameter of *ca*. 2‍ ‍mm). After an overnight incubation at 20°C, gel beads were washed with fresh inorganic medium. The gel beads obtained were inoculated into the up-flow column reactors.

### Chemical ana­lysis

NH_4_^+^, NO_2_^–^, and NO_3_^–^ concentrations were measured using the ion chromatograph IC-2010 equipped with the TSKgel SuperIC-Anion HS or TSKgel SuperIC-Cation HS column (Tosoh). NH_2_OH concentrations were measured colorimetrically (Frear and Burrell, 1955). Briefly, liquid samples were mixed with 0.48% (w/v) trichloroacetic acid, 0.2% (w/v) 8-hydroxyquinoline, and 0.2 M Na_2_CO_3_, heated at 100°C for 1‍ ‍min, and absorbance was then measured at a wavelength of 705‍ ‍nm using the spectrophotometer V-630bio (Jasco). N_2_H_4_ concentrations were measured colorimetrically using *p*-dimethyl-aminobenzaldehyde (Watt and Chrisp, 1952). Briefly, liquid samples were mixed with 0.12 M *p*-dimethyl-aminobenzaldehyde, and absorbance was measured at a wavelength of 460‍ ‍nm.

^14-15^N_2_ and ^15-15^N_2_ concentrations were measured by gas chromatography mass spectrometry (GC/MS) (Isobe *et al.*, 2011a; 2011b). Fifty microliters of the headspace gas was collected using a 100-‍μL gas-tight glass syringe and immediately injected into the gas chromatograph GCMS-QP 2010 SE (Shimadzu) equipped with a fused silica capillary column (Agilent Technologies). Peaks at *m/z*=29 and 30 corresponding to ^14-15^N_2_ and ^15-15^N_2_ were monitored, and concentrations were calculated using a standard curve prepared using ^15-15^N_2_ gas (Cambridge Isotope Laboratories). The ^14-15^N_2_ and ^15-15^N_2_ concentrations of ^15^NH_2_OH were calculated by considering the ^15^N enrichment of ^15^NH_2_OH·HCl (^15^N, 98%) and the natural abundance of ^14-15^N_2_ and ^15-15^N_2_ in atmospheric N_2_ gas contaminated at the injection of the gas sample.

### qPCR assay

The copy numbers of the anammox bacterial 16S rRNA gene were measured using a qPCR assay. Genomic DNA was extracted from gel beads collected from the up-flow column reactors using the FastDNA SPIN kit (Qiagen) according to the instruction manual supplied by the manufacturer. The qPCR assay was conducted using the ABI7500 fast Real-Time PCR System (Thermo Fisher Scientific) and Premix Ex Taq (Probe qPCR) (TakaraBio) under previously described thermal conditions (Zhang and Okabe, 2017a). The oligonucleotide primers and TaqMan probe used for the PCR amplification of the *B. sinica* 16S rRNA gene were BRS95F, BRS170R, and BRS130P. Standard curves (10^1^ to 10^6^ copies μL^–1^) were prepared using a dilution series of plasmid DNAs containing a partial *B. sinica* 16S rRNA gene sequence.

## Results

### NH_2_OH disproportionation by phylogenetically different anammox bacteria

**Fig. 1.**
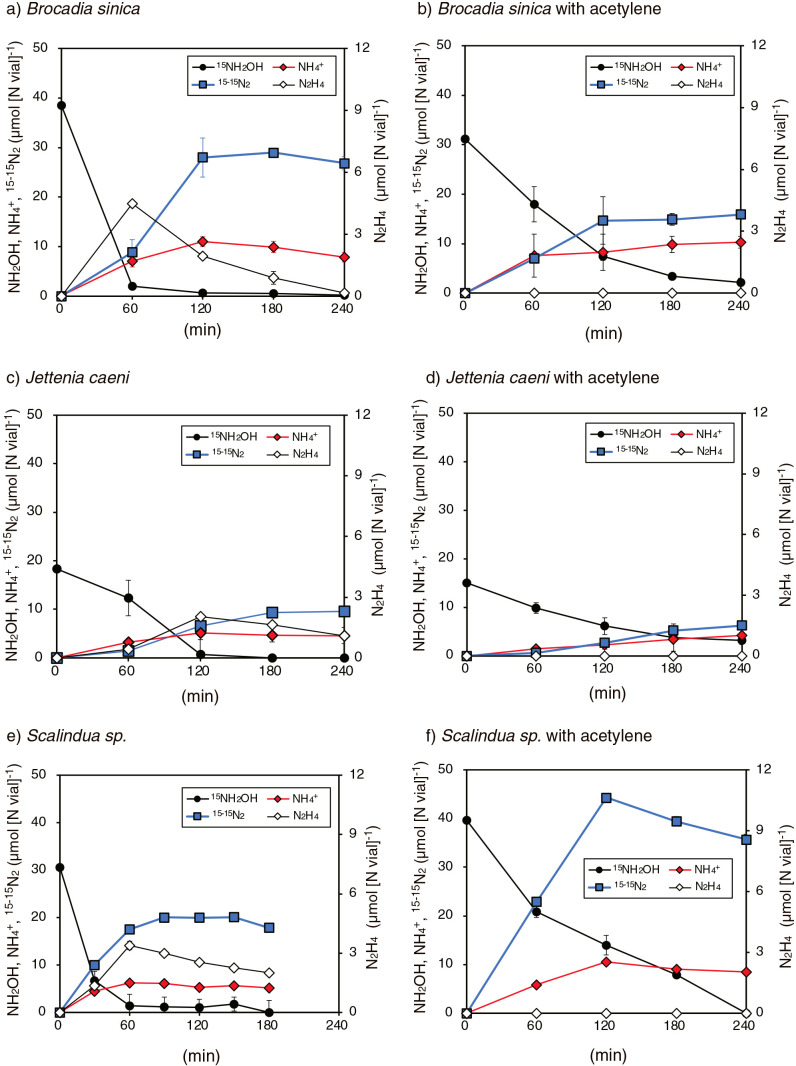
^15^NH_2_OH-tracing batch incubations to demonstrate anammox bacterial NH_2_OH disproportionation. Twenty-five milliliters of *Brocadia sinica* (panel a and b), *Jettenia caeni* (panel c and d), and *Scalindua* sp. (panel e and f) cultures were incubated with the addition of 2.5‍ ‍mM ^15^NH_2_OH in 70-mL glass vials. Incubations were repeated with the addition of 30‍ ‍μM acetylene (panel b, d, and f). All incubations were performed in triplicate, and symbols and error bars represent mean values and the range of standard deviations, respectively. The standard deviations of data points are often within the symbols.

Anammox bacterial cells of *B. sinica*, *J. caeni*, and *Scalindua sp.* were incubated with the addition of 1.0 to 2.5‍ ‍mM ^15^NH_2_OH. As shown in Fig. 1a, c, and e, ^15^NH_2_OH consumption occurred in the culture concurrently with the production of ^15-15^N_2_ gas and NH_4_^+^. N_2_H_4_ was also produced and markedly increased when NH_2_OH concentrations decreased below 0.1‍ ‍mM (*i.e.*, a 60-min incubation for *B. sinica* and *Scalindua* sp. and a 120-min incubation for *J. caeni*). ^14-15^N_2_, NO_2_^–^, and NO_3_^–^ were not detected during any incubations, and ^15^NH_2_OH consumption was negligible in vials without anammox bacterial cells (*i.e.*, abiotic control incubation).

Batch incubations were repeated with the addition of 30‍ ‍μM acetylene. Anammox bacterial cells consumed ^15^NH_2_OH (Fig. 1b, d, and f), whereas consumption rates were >3-fold lower than those without acetylene. Although ^15-15^N_2_ gas and NH_4_^+^ were produced during the incubation, N_2_H_4_ was not produced in any anammox bacterial cultures.

**Fig. 2.**
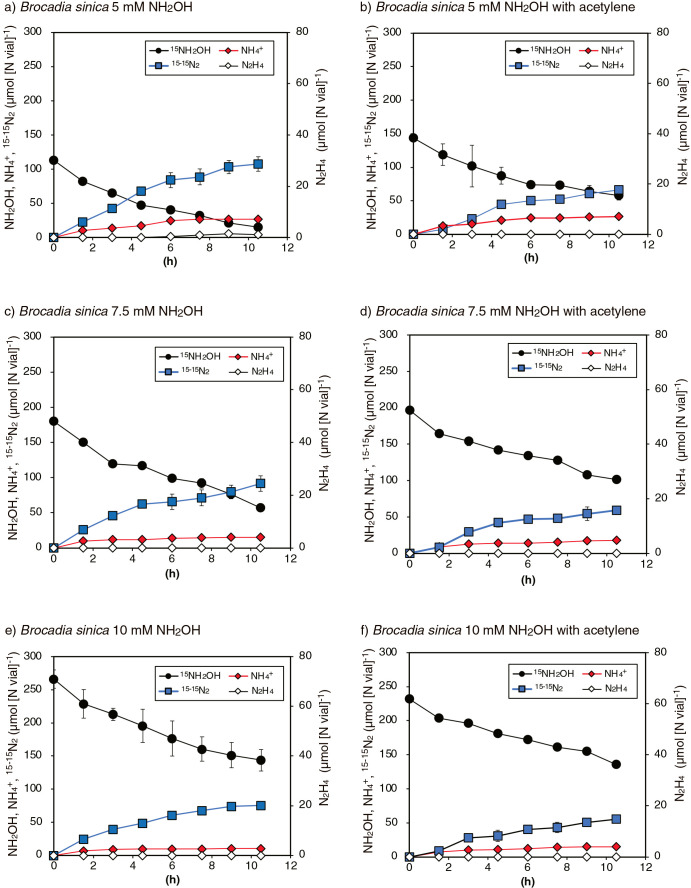
Effects of the initial NH_2_OH concentration on NH_2_OH disproportionation by *Brocadia sinica*. Twenty-five milliliters of the *Brocadia sinica* culture was incubated with the addition of 5‍ ‍mM (panel a and b), 7.5‍ ‍mM (panel c and d), and 10‍ ‍mM ^15^NH_2_OH (panel e and f) in 70-mL glass vials. Incubations were repeated with the addition of 30‍ ‍μM acetylene (panel b, d, and f). All incubations were performed in triplicate, and symbols and error bars represent mean values and the range of standard deviations, respectively. The standard deviations of data points are often within the symbols.

The above batch incubation of *B. sinica* was repeated with an increase in the initial NH_2_OH concentration (from 5 to 10‍ ‍mM) with/without 30‍ ‍μM acetylene. As shown in Fig. 2, ^15^NH_2_OH consumption and the concomitant production of ^15-15^N_2_ and NH_4_^+^ occurred, similar to the batch incubation with the addition of 2.5‍ ‍mM ^15^NH_2_OH (*i.e.*, Fig. 1a and b). It is important to note that N_2_H_4_ production only occurred when NH_2_OH concentrations decreased below 1.5‍ ‍mM (Fig. 2a after 7.5 h of the incubation), and N_2_H_4_ production was not observed in Fig. 2c and e.

The stoichiometry and nitrogen mass balance of the above batch incubations are shown in Table 1. The theoretical values for Δ^15-15^N_2_/Δ^15^NH_2_OH and Δ^15^NH_4_^+^/Δ^15^NH_2_OH were 0.33 and 0.33, respectively when NH_2_OH disproportionation occurred according to eq. 2. The values for Δ^15-15^N_2_/Δ^15^NH_2_OH and Δ^15^NH_4_^+^/Δ^15^NH_2_OH obtained from batch incubations with the addition of 2.5 or 5‍ ‍mM ^15^NH_2_OH were in the range of 0.19–0.45 and 0.17–0.36, respectively. These values were generally close to the theoretical values and those for Δ^15^NH_4_^+^/Δ^15^NH_2_OH in the *K. stuttgartiensis* culture (0.19–0.48), except for the following batch incubations: *B. sinica* with 2.5‍ ‍mM ^15^NH_2_OH without acetylene (0.2 for Δ^15^NH_4_^+^/Δ^15^NH_2_OH), *B. sinica* with 5‍ ‍mM ^15^NH_2_OH with acetylene (0.19 for Δ^15-15^N_2_/Δ^15^NH_2_OH), and *Scalindua* sp. with 2.5‍ ‍mM ^15^NH_2_OH with and without acetylene (0.17 or 0.21 for Δ^15^NH_4_^+^/Δ^15^NH_2_OH). Batch incubations of *B. sinica* with 7.5 or 10‍ ‍mM NH_2_OH generally resulted in lower values for Δ^15-15^N_2_/Δ^15^NH_2_OH and Δ^15^NH_4_^+^/Δ^15^NH_2_OH, which were in the ranges of 0.14–0.19 and 0.09–0.19, respectively.

### Cultivation of B. sinica with NH_2_OH in up-flow column reactors

**Fig. 3.**
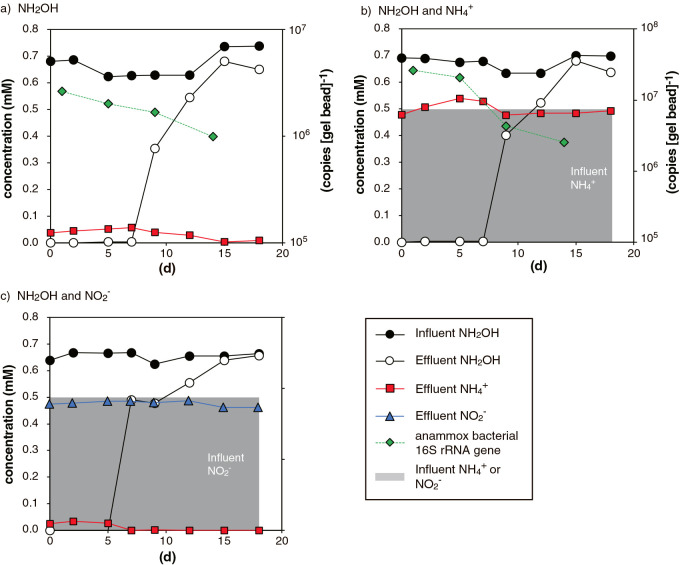
NH_2_OH consumption by *Brocadia sinica* in continuous up-flow column reactors. Up-flow column reactors were inoculated with PVA-SA gel beads immobilizing *B. sinica* cells and were operated with the continuous feeding of NH_2_OH (panel a), NH_2_OH and NH_4_^+^ (panel b), and NH_2_OH and NO_2_^–^ (panel c).

NH_2_OH consumption and the abundance of the anammox bacterial 16S rRNA gene were examined in the 1) NH_2_OH-, 2) NH_2_OH and NH_4_^+^-, and 3) NH_2_OH and NO_2_^–^-feeding up-flow column reactors. In all operated reactors, NH_2_OH consumption markedly decreased after 7‍ ‍d of operation, and halted after 15‍ ‍d of operation (Fig. 3). NH_4_^+^ concentrations in the effluents of the NH_2_OH- and NH_2_OH and NO_2_^–^-feeding reactors were in the same range (Fig. 3a and c, respectively), and the consumption of NH_4_^+^ did not occur in the NH_2_OH and NH_4_^+^-feeding reactor (Fig. 3b). The copy numbers of the anammox bacterial 16S rRNA gene decreased over time, and its abundance after 14‍ ‍d of operation was an order of magnitude less than that after 1‍ ‍d of operation. Copy numbers were not measured in the reactor fed with NH_2_OH and NO_2_^–^ (Fig. 3c).

## Discussion

NH_2_OH disproportionation was examined using phylogenetically different anammox bacteria, and the present study clearly indicated that NH_2_OH disproportionation is a common nitrogen transformation process of anammox bacteria (Table 1). Although previous studies (van der Star *et al.*, 2008; Soler-Jofra *et al.*, 2020) reported anammox bacterial NH_2_OH disproportionation, they did not examine the amount of N_2_ gas produced. The present study performed sophisticated ^15^NH_2_OH-tracing batch incubations, and revealed that anammox bacteria yielded both N_2_ gas and NH_4_^+^ from NH_2_OH disproportionation. ^15^NH_2_OH-tracing batch incubations enabled the stoichiometry of ^15-15^N_2_ and ^15^NH_4_^+^ production to ^15^NH_2_OH consumption to be examined (Table 1), and the results obtained indicated that an increase in the initial concentration of NH_2_OH resulted in a decrease in Δ^15-15^N_2_/Δ^15^NH_2_OH and Δ^15^NH_4_^+^/Δ^15^NH_2_OH for *B. sinica*. Furthermore, the addition of acetylene resulted in a decrease and increase in Δ^15-15^N_2_/Δ^15^NH_2_OH and Δ^15^NH_4_/Δ^15^NH_2_OH, respectively. These variations in stoichiometry suggested that ^15^N_2_ and/or ^15^NH_4_^+^ were produced by multiple nitrogen transformation pathways other than NH_2_OH disproportionation. Anammox bacterial hydroxylamine dehydrogenase oxidizes NH_2_OH to NO (Maalcke *et al.*, 2014), and N_2_ gas may be produced using the NO formed by the anammox process (the coupling of NH_4_^+^ and NO) (Kartal *et al.*, 2013). Although the reduction of NH_2_OH to NH_4_^+^ by anammox bacterial cells has not yet been demonstrated, anammox bacteria are capable of dissimilatory nitrite reduction to ammonium (Kartal *et al.*, 2007).

N_2_ and NH_4_^+^-forming NH_2_OH disproportionation has not yet been reported for microorganisms other than anammox bacteria. It requires the formation of a nitrogen-nitrogen bond for N_2_ gas, and only nitric oxide reductase and hydrazine synthase catalyze this reaction (Dietl *et al.*, 2015). This may be the reason why N_2_ and NH_4_^+^-forming NH_2_OH disproportionation has not yet been detected in microbial cultures other than anammox bacteria. Apart from N_2_ and NH_4_^+^-forming NH_2_OH disproportionation, 1) NH_4_^+^ and NO-, 2) NH_4_^+^ and NO_2_^–^-, and 3) N_2_O and NH_4_^+^-forming NH_2_OH disproportionation are thermodynamically favorable (Pacheco *et al.*, 2011); however, limited information is currently available on their activities or involvement in the nitrogen transformation process in natural and man-made ecosystems.

The addition of acetylene did not induce the accumulation of N_2_H_4_, but reduced NH_2_OH consumption (Fig. 1b, d, and f), which indicated that acetylene inhibited the enzymes involved in both NH_2_OH consumption and N_2_H_4_ production reactions. *B. sinica* hydrazine synthase utilizes NH_2_OH as a substrate for N_2_H_4_ synthesis (Oshiki *et al.*, 2016a); therefore, hydrazine synthase may be the enzyme that is inhibited by acetylene. The inhibition of anammox bacterial activity by acetylene has been reported (Jensen *et al.*, 2007); however, the underlying mechanism(s) remain unknown. Apart from hydrazine synthase, acetylene inhibited the NO_2_^–^ reduction reaction of anammox bacteria (Kartal *et al.*, 2011; Oshiki *et al.*, 2016a), indicating that acetylene suppresses multiple nitrogen transformation reactions of anammox bacteria. Although the acetylene inhibition of copper-containing metalloproteins, such as ammonia monooxygenase and nitrous oxide reductase (Amo and Nos, respectively) has been described (Ensign *et al.*, 1993; Gilch *et al.*, 2009), the involvement of copper-containing metalloproteins in anammox bacterial metabolism, particularly N_2_H_4_ production, remains unclear. The binding site of acetylene to anammox bacterial enzymes is of interest for obtaining a more detailed understanding of the acetylene inhibition of anammox bacteria. The addition of 30‍ ‍μM acetylene did not completely inhibit the production of ^15-15^N_2_ gas or NH_4_^+^ (Fig. 1 and 2). This result suggests the incomplete inhibition of anammox bacterial hydrazine synthase with the addition of 30‍ ‍μM acetylene (Jensen *et al.*, 2007) and/or the production of ^15-15^N_2_ gas and NH_4_^+^, but not through N_2_H_4_. The corresponding mechanism has not yet been elucidated, and a further understanding of acetylene inhibition by anammox bacteria will provide novel insights.

Apart from the above acetylene inhibition, high NH_2_OH concentrations (>0.1‍ ‍mM NH_2_OH in Fig 1a, c, and e, and >1.5‍ ‍mM NH_2_OH in Fig. 2a) did not induce the accumulation of N_2_H_4_. This behavior cannot simply be explained by the inhibition of anammox bacterial hydrazine dehydrogenase involved in N_2_H_4_ oxidation to N_2_. The N_2_H_4_ oxidation activities of purified anammox bacterial hydrazine dehydrogenase were inhibited in the presence of 2.4 to 7.9‍ ‍μM NH_2_OH (Shimamura *et al.*, 2007; Maalcke *et al.*, 2016), and this inhibition provides an explanation for the accumulation of N_2_H_4_ during batch incubations, but not for the lack of its accumulation at high NH_2_OH concentrations. On the other hand, anammox bacterial N_2_H_4_ accumulation may be explained by the balance between N_2_H_4_ production and consumption reactions (eq. 3 and 4, respectively), as reported in previous studies (van der Star *et al.*, 2008; Soler-Jofra *et al.*, 2020). N_2_H_4_ production and consumption reactions require 1 and 2 moles of NH_2_OH, respectively, and higher NH_2_OH concentrations increase N_2_H_4_ consumption rates more than N_2_H_4_ production rates; therefore, N_2_H_4_ does not accumulate at high NH_2_OH concentrations. The affinity constants of eq. 3 and 4 for NH_2_OH need to be examined in more detail in order to clarify the N_2_H_4_ accumulation behavior of anammox bacterial cells.

The results of the up-flow column reactor experiments revealed that *B. sinica* did not proliferate with NH_2_OH disproportion (Fig. 3), and this is the first experimental evidence to show that an anammox culture cannot be maintained with NH_2_OH as the sole energy source. A 1 log reduction in the *B. sinica* 16S rRNA gene copy number clearly indicated that *B. sinica* did not proliferate in the operated reactors. *B. sinica* cells may have been compromised during reactor operation due to the high toxicity and mutagenesis of NH_2_OH, which resulted in a decrease in the *B. sinica* 16S rRNA gene copy number in PVA-SA gel beads. We previously reported a log reduction in the anammox bacterial 16S rRNA gene copy number in PVA-SA gel beads under unfavorable cultivation conditions (Zhang *et al.*, 2017b). It is important to note that *B. sinica* cells preferentially performed NH_2_OH disproportionation over anammox. Anammox using NO_2_^–^ or NH_2_OH did not occur in the NH_2_OH and NO_2_^–^- or NH_2_OH and NH_4_^+^-feeding reactors (Fig. 3c and b, respectively), which indicated that the anammox activities of *B. sinica* were inhibited in these reactors by NH_2_OH. Therefore, low NH_2_OH concentrations need to be maintained in order to avoid the inhibition of anammox activities and achieve the stable performance of nitrogen removal. Although the inhibitory effects of NH_2_OH on nitrifying bacteria have been investigated (Kindaichi *et al.*, 2004; Soler-Jofra *et al.*, 2021), the IC_50_ concentration of NH_2_OH for anammox bacteria has not yet been systematically examined. In the present study, the sudden deterioration of NH_2_OH consumption occurred after 7‍ ‍d of operation; however, the concentration of NH_2_OH in the influent was markedly lower than those in batch incubations in which the marked deterioration of NH_2_OH consumption did not occur (*i.e.*, 0.7 and 2.5–10‍ ‍mM, respectively). Therefore, it was not possible to approximate the IC_50_ concentration of NH_2_OH inhibition from short-term batch incubations, and further studies are warranted to assess the IC_50_ concentration of the long-term inhibition of NH_2_OH. NH_2_OH may accumulate at the submicromolar range in natural aquatic environments (Fukumori *et al.*, 2003; Bikbulatova *et al.*, 2007) and at the submillimolar range in nitrifying cultures (Soler-Jofra *et al.*, 2021 and references therein); however, the impact of NH_2_OH on nitrogen transformation reactions remains unclear. Further studies are needed to examine NH_2_OH transformation, including anammox bacterial NH_2_OH disproportionation, in natural and man-made ecosystems.

## Citation

Oshiki, M., Gao, L., Zhang, L., and Okabe, S. (2022) NH_2_OH Disproportionation Mediated by Anaerobic Ammonium-oxidizing (Anammox) Bacteria. *Microbes Environ ***37**: ME21092.

https://doi.org/10.1264/jsme2.ME21092

## Figures and Tables

**Table 1.** Stoichiometry of NH_2_OH disproportionation mediated by anammox bacteria. The values obtained in the present study were mean values from triplicate biological replicates, and values were calculated from the initial and final concentrations during batch incubations. The N-mass balance (%) was calculated by dividing the total amounts of ^15^NH_2_OH, ^15^NH_4_^+^, and ^15-15^N_2_ at the end of the incubation (μmol [N vial]^–1^) by the initial amounts of ^15^NH_2_OH (μmol [N vial]^–1^).

Species	NH_2_OH (mM)	Acetylene*	N-mass balance	Δ^15-15^N_2_/ΔNH_2_OH (mol/mol)	Δ^15^NH_4_^+^/ΔNH_2_OH (mol/mol)	References
*B. sinica*	2.5	w/o	91%	0.35	0.20	This study
	5	w/o	85%	0.28	0.27	
	7.5	w/o	66%	0.19	0.12	
	10	w/o	72%	0.16	0.09	
	2	+	91%	0.27	0.35	
	5	+	81%	0.19	0.30	
	7.5	+	76%	0.16	0.19	
	10	+	77%	0.14	0.15	
*J. caeni*	1	w/o	83%	0.26	0.25	
		+	91%	0.26	0.36	
*Scalindua sp.*	2	w/o	82%	0.29	0.17	
	2.5	+	111%	0.45	0.21	
*K. stuttgartiensis*	1.6–10	w/o	NA	ND	0.19–0.48	van der Star *et al.*, 2008 Soler-Jofra *et al.*, 2020
*B. flugida*	4	w/o	NA	ND	0.25	van der Star *et al.*, 2008

*; w/o and +: without and with the addition of 30‍ ‍μM acetylene, respectively, NA: not available, ND: not determined.
